# Supplementary material for: Disharmonic Inflammatory Signatures in COVID-19: Augmented Neutrophils’ but Impaired Monocytes’ and Dendritic Cells’ Responsiveness
Source: Cells. 2020 Sep 29;9(10):2206. doi: 10.3390/cells9102206 (PMC7600406; doi:10.3390/cells9102206)

Supplementary Figure 1: A. Peripheral neutrophil phenotype of COVID-19 patients diversified according to the disease severity. **B.** Principal component analysis of COVID-19 patients' and HD neutrophil phenotype. **C.** Representative histograms of a phenotype of HD and COVID-19 patients' neutrophils upon 1μg/ml LPS and 10μg/ml ssRNA stimulation in different time points. **D.** Immature neutrophil phenotype of COVID-19 patients (n=19) upon the hospital admission and HD (n=29). **E.** Serum level of G-CSF in COVID-19 patients (n=17) and HD (n=25) detected by ELISA. **F.** Western blot analysis of overall tyrosine phosphorylation, pSTAT3, pSTAT1, pJNK, pp38 and pIRF3 in neutrophil extracts of COVID-19 (n=5) and HD (n=5). Band area values were used for semi-quantification. Graphs are expressed as a ratio of band area value oa analyzed protein/beta actin using ImageJ software. Statistical analysis was performed using Mann-Whitney unpaired *t*-test. Values of p<0.05 (*), p<0.01 (**), p<0.001 (***) and p<0.0001 (****) were considered statistically significant.

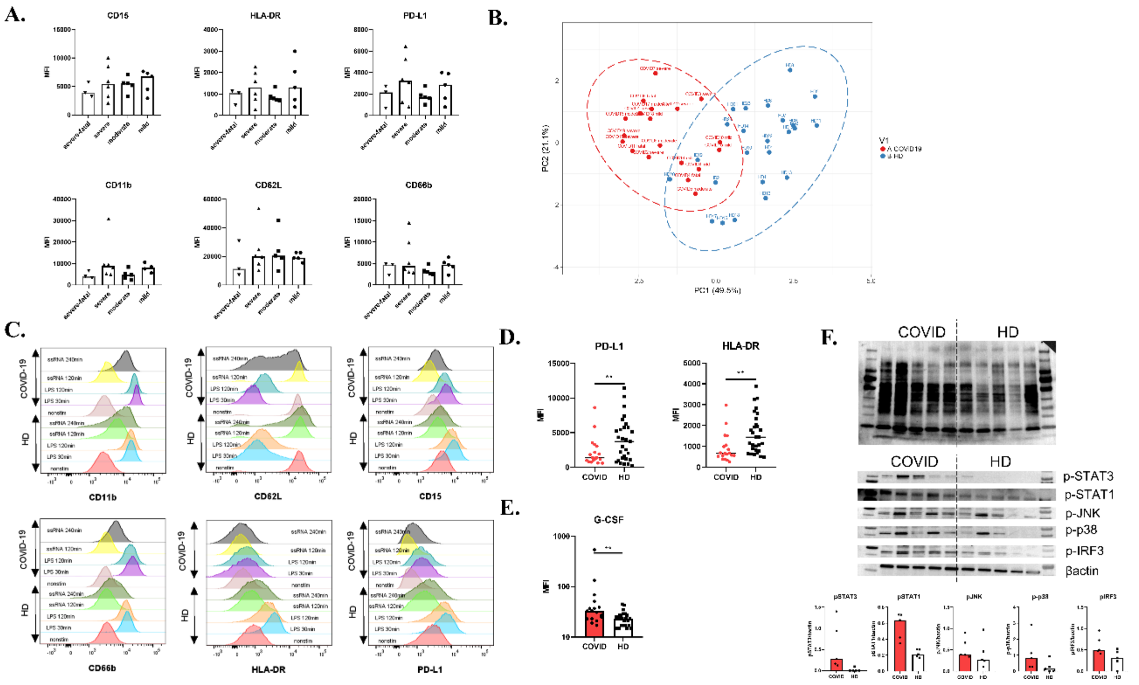

Supplementary Figure 2: A. PBMCs from COVID-19 patients (n=13) and HD (n=13) were stimulated overnight with 10µg/ml ssRNA, 1µg/ml R848 and 50µg/ml polyI:C or left untreated, and the production of IL-6, IL-1ß and TNFα was analysed with LUMINEX. **B.** *IFNAR* expression in PBMCs and isolated neutrophils was analysed by RT-PCR and normalized to *GAPDH* expression. **C.** Basal TNFα, IL-1β and IL-6 expression in monocytes, mDCs and pDCs detected in COVID-19 (n=7) and HD (n=6) by flow cytometry. Data are expressed as MFI. Statistical analysis was performed using Mann-Whitney unpaired *t*-test. Values of p<0.05 (*), p<0.01 (**), p<0.001 (***) and p<0.0001 (****) were considered statistically significant.

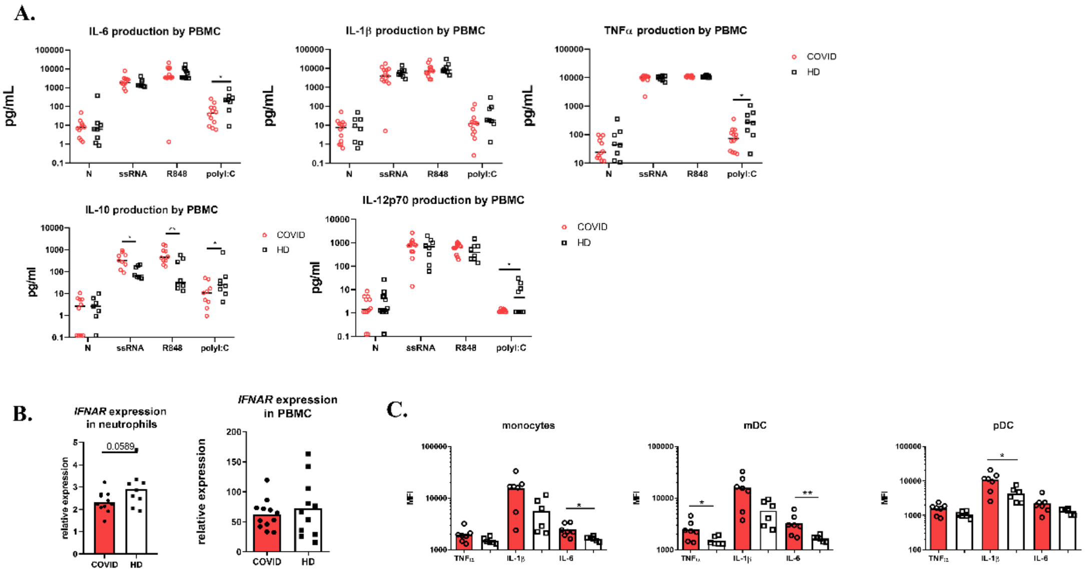

Supplement: Supplementary file 1 [file cells-09-02206-s001.pdf]
